# Functionalized TiO_2_ nanoparticles by single-step hydrothermal synthesis: the role of the silane coupling agents

**DOI:** 10.3762/bjnano.8.33

**Published:** 2017-01-31

**Authors:** Antoine R M Dalod, Lars Henriksen, Tor Grande, Mari-Ann Einarsrud

**Affiliations:** 1 Department of Materials Science and Engineering, NTNU, Norwegian University of Science and Technology, NO-7491 Trondheim, Norway; 2 poLight AS, Kongeveien 77, NO-3188 Horten, Norway

**Keywords:** core–shell nanoparticles, functionalized nanoparticles, hydrothermal synthesis, oriented attachment, silane coupling agent

## Abstract

A simple, robust and versatile hydrothermal synthesis route to in situ functionalized TiO_2_ nanoparticles was developed using titanium(IV) isopropoxide as Ti-precursor and selected silane coupling agents (3-aminopropyltriethoxysilane (APTES), 3-(2-aminoethylamino)propyldimethoxymethylsilane (AEAPS), and *n*-decyltriethoxysilane (DTES)). Spherical nanoparticles (ca. 9 nm) with narrow size distribution were obtained by using DTES or by synthesis performed without silane coupling agents. Rod-like nanoparticles along with 9 nm spherical nanoparticles were formed using aminosilane coupling agents because of a combination of oriented attachment of nanoparticles and specific adsorption of the aminosilane on crystallographic faces of anatase nanoparticles. The nanoparticles were functionalized in situ and became hydrophobic as silanes reacted to form covalent bonds on the surface of TiO_2_. The versatility of the aqueous synthesis route was demonstrated, and by selecting the type of silane coupling agent the surface properties of the TiO_2_ nanoparticles could be tailored. This synthesis route has been further developed into a two-step synthesis to TiO_2_–SiO_2_ core–shell nanoparticles. Combustion of the silane coupling agents up to 700 °C leads to the formation of a nanometric amorphous SiO_2_ layer, preventing growth and phase transition of the in situ functionalized nanoparticles.

## Introduction

Because of the high surface-to-volume ratio, the intrinsic properties of titanium dioxide (TiO_2_) nanoparticles have led to exploitation in many fields such as in photocatalysis [1], solar cells [2], and in biomedical applications [3]. The naturally occurring phases of TiO_2_ are rutile (thermodynamically stable polymorph), brookite, and anatase [4]. Due to the differences in surface energy, anatase and brookite are more stable than rutile at nanosize, and anatase is more stable than brookite at even smaller sizes (generally below 15–30 nm) [5–7]. Surface modification of TiO_2_ nanoparticles, via core–shell structures or grafted nanoparticles [8], has resulted in new applications such as nanofiller for polymer nanocomposites [9–10], coatings [11], and biosensors [3,12]. Classical synthesis routes for surface-functionalized particles are following two steps: particles synthesis followed by a post-functionalization process [9–10,13].

Post-functionalization of TiO_2_ nanoparticles with silane coupling agents was obtained via reflux in aqueous solution [14–15]. Chen et al. investigated interactions of 3-aminopropyltrimethoxysilane (APTMS) and phenyltrimethoxysilane with commercially available TiO_2_ nanoparticles (Degussa P-25) [14]. They concluded that the silane coupling agents covalently bond onto the surface of TiO_2_ nanoparticles. Using a mixture of isomeric octyltriethoxysilanes (OTES), Milanesi et al. focused on the structure of the hydrophobic layer and proposed that cross-linking (via Si–O–Si bonds) and chemical bonding (via Ti–O–Si bonds) of silanes onto TiO_2_ nanoparticles occurred [16]. Later, Zhao et al. detailed the cross-linking and chemical bonding mechanisms of APTMS and 3-isocyanatopropyltrimethoxysilane on TiO_2_ nanoparticles [15]. A contact angle of about 150° for water was measured demonstrating hydrophobic nanoparticles. Wang et al. functionalized commercial TiO_2_ nanoparticles in aqueous solution via ultrasonic treatment at room temperature with 3-(trimethoxysilyl)propyl methacrylate [17]. The resulting particles exhibited hydrophobic behavior. Another study reported room-temperature surface functionalization of commercial TiO_2_ nanoparticles in ethanol using *n*-(6-aminohexyl)aminopropyltrimethoxysilane [18].

Nanoparticle synthesis with in situ surface functionalization has the advantage to reduce the number of reaction steps and is thus of greater interest for potential industrial applications. Teleki et al. developed a route for the continuous production of surface-functionalized TiO_2_ via flame spray pyrolysis where the particles were directly functionalized after synthesis with OTES [19]. Depending on the conditions, they obtained surface-functionalized TiO_2_ nanoparticles with an average size of 40 nm and they determined a maximum surface coverage of about 2.6 OTES molecules per square nanometer. Niederberger et al. developed a room-temperature non-aqueous in situ functionalization process of TiO_2_ nanoparticles with 4-*tert*-butylcatechol and dopamine [20]. A brittle brown solid and a dark red powder was obtained for 4-*tert*-butylcatechol and dopamine surface functionalized samples, respectively. More recently, Gao and Cui reported a sol–gel method in which TiO_2_ nanoparticles functionalized with chlorinated alcohols through hydrogen bonding were produced [21]. However, sol–gel synthesis often leads to poorly crystalline particles [22].

Hydrothermal synthesis [23] is simple and cost efficient [24] and allows for improved crystallinity compared to sol–gel methods [22] giving improved TiO_2_ characteristics for applications such as photocatalysis and solar cell applications [2,25–26]. Typically used precursors are titanium alkoxides where the formation of anatase nanocrystals occurs through hydrolysis and condensation [22]. To our knowledge there is only one work where in situ functionalization of TiO_2_ nanoparticles using solvothermal synthesis is reported. Koziej et al. used trimethoxy(7-octen-1-yl)silane (7-OTS) and 3-(trimethoxysilyl)propyl methacrylate coupling agents during TiO_2_ nanoparticle synthesis from titanium isopropoxide in anhydrous benzyl alcohol [27]. The particles however needed further post functionalization with 7-OTS for better compatibility with organic solvent and PMMA.

Here, we report on a novel and versatile in situ aqueous hydrothermal synthesis route to surface-functionalized TiO_2_ nanoparticles using selected silane coupling agents. The nanoparticles were characterized with respect to crystal structure, size, size distribution, specific surface area, surface coverage, and hydrophobicity. Tuning the surface properties of the nanoparticles for different applications by selecting the silane coupling agent is discussed. We further report the effect of heat treatment of the nanoparticles for the formation of core–shell TiO_2_–SiO_2_ nanoparticles.

## Experimental

### Synthesis

The synthesis of the non-functionalized TiO_2_ nanoparticles was based on a hydrothermal route previously described by Hayashi and Torii, using titanium(IV) isopropoxide (TIP) as precursor [28]. The synthesis method was further developed for in situ surface functionalization using selected silane coupling agents: 3-aminopropyltriethoxysilane (Sigma-Aldrich, 99%), 3-(2-aminoethylamino)propyldimethoxymethylsilane (Fluka, ≥95%), and *n*-decyltriethoxysilane (ABCR, 97%); abbreviated APTES, AEAPS, and DTES, respectively.

TIP (28 mmol, Sigma-Aldrich, ≥97%) was mixed with distilled water, to which the silane coupling agent (TIP/silane molar ratio equal to 10:1) was initially added to give a filling factor of 70% in the autoclave. The solutions were vigorously stirred for 10 min prior to transfer into a PTFE-lined autoclave (Parr, 125 mL) and heated for 2 h at 200 °C. After cooling to room temperature, the products were centrifuged (10000 rpm, 10 min) and washed with distilled water. This process was repeated three times. The obtained slurries were dried for about 12 h at 100 °C for analysis.

TiO_2_ samples in situ surface-functionalized with APTES, AEAPS, and DTES are labeled Ti-APTES, Ti-AEAPS, and Ti-DTES, respectively. Heat-treated samples at 700 °C in synthetic air during thermogravimetric analysis (see details below) were further investigated and are labeled adding the suffix “-HT” to the original sample name, i.e., TiO_2_-HT, Ti-APTES-HT, Ti-AEAPS-HT, and Ti-DTES-HT.

### Characterization

Powder X-ray diffraction (XRD) was performed on a Bruker D8 Advance DAVINCI working in Bragg–Brentano (θ/2θ) geometry. Diffractograms were recorded under Cu Kα radiation, with a step size of 0.013°, an integration time of 0.4 s, and using variable divergent slits. Rietveld refinements and crystallite sizes were obtained using TOPAS (Bruker AXS version 4.2).

Scanning electron microscopy (SEM) images were recorded on an in-lens cold-field-emission S(T)EM Hitachi S-5500. The acceleration voltage was set at 7 kV and secondary electrons were detected. For the preparation of the samples, a drop of particles in water obtained after the centrifugation steps was placed on an aluminum sample holder which was set to dry overnight. The line-intercept method was used to calculate average particle sizes, using sample pictures containing more than 300 intercepts.

Transmission electron microscopy (TEM) images were recorded on a JEOL 2100 equipped with Oxford X-Max 80 SDD detector for energy-dispersive X-ray spectroscopy (EDS) analysis. The acceleration voltage was set at 200 kV. For the preparation of the samples, the nanoparticles were dispersed in anhydrous 2-propanol (Sigma-Aldrich, 99.5%) by sonication for 15 min. A droplet of the suspension was then placed on a carbon-coated copper TEM grid, which was set to rest until evaporation of the solvent. The *d_hkl_* distances were measured by extracting an area of interest from the HR-TEM images with fast Fourier transform analysis, and calculating the average distance over more than ten consecutive *hkl* planes, using DigitalMicrograph (Gatan Inc. version 3.01).

Specific surface area (BET method [29]) and pore size distribution (BJH method [30]) were measured by nitrogen adsorption on a Micrometrics Tristar 3000. Samples were degassed for 12 h at 180 °C in vacuum prior to analysis. Particle sizes were estimated from the surface area assuming non-porous and spherical particles.

Fourier-transform infrared (FTIR) spectra were acquired on a Bruker Vertex 80v FTIR equipped with Bruker Platinum ATR diamond system from 400 to 4000 cm^−1^, under medium vacuum (280 Pa). A background was collected under medium vacuum, without sample. Between each analysis, the ATR diamond was cleaned with isopropanol, for which vacuum provides fast evaporation and no specific adsorption bands of isopropanol were observed. A total of 128 scans were acquired for each sample at a resolution of 1 cm^−1^.

Thermogravimetric analysis (TGA) was acquired on a Netzsch Jupiter STA 449 C using an alumina crucible. The nanoparticles were firstly heat-treated from 25 to 150 °C (10 °C·min^−1^), maintained at 150 °C for 30 min, cooled down to room temperature, and heat-treated again from 25 to 200 °C (2 °C·min^−1^) in order to remove adsorbed water. The samples were finally heat-treated from 100 to 700 °C (2 °C·min^−1^). All treatments were performed under synthetic air.

## Results and Discussion

### Structure, particle size and morphology

**Figure 1:**
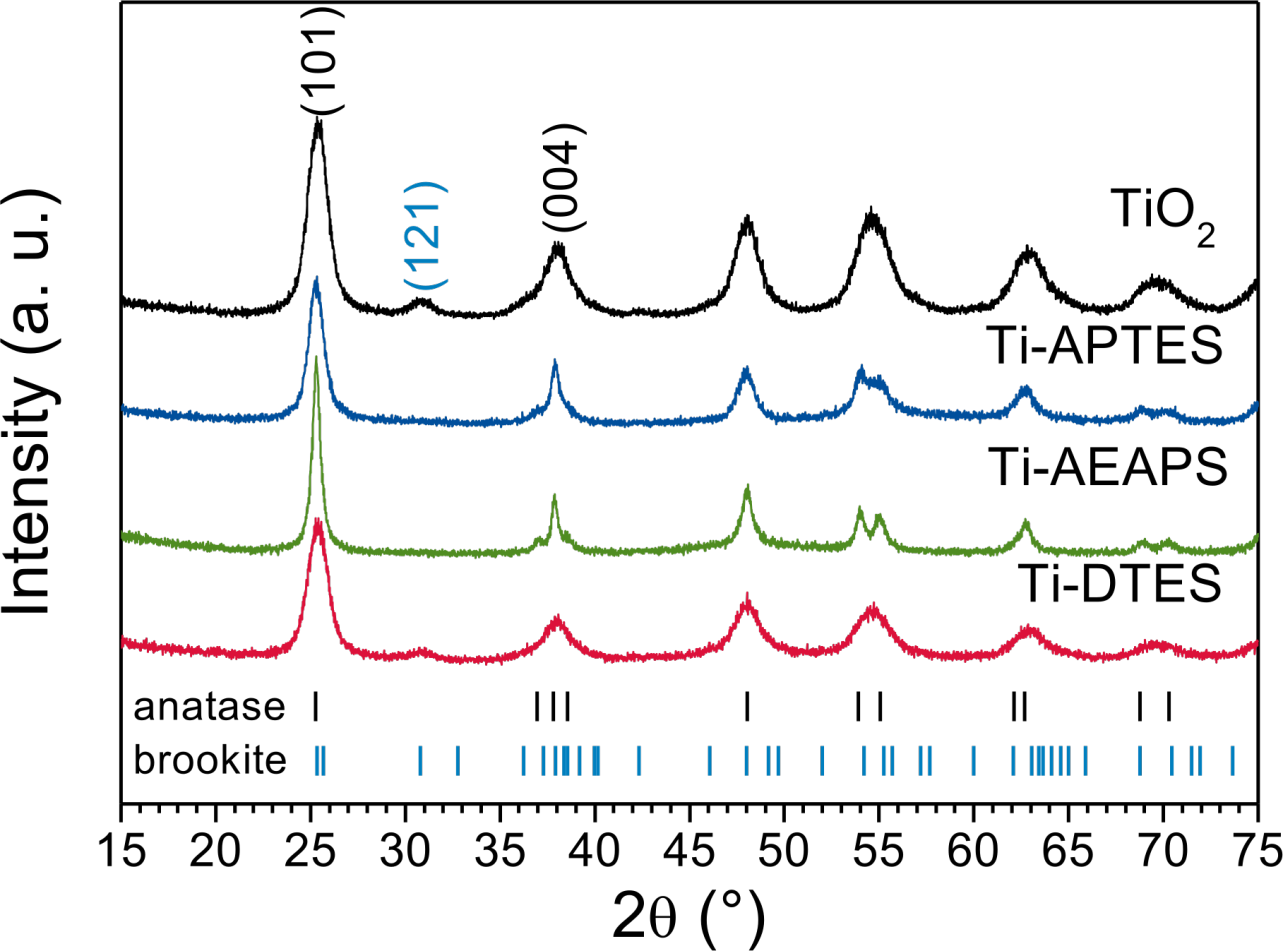
XRD patterns of TiO_2_ and in situ surface-functionalized TiO_2_ nanoparticles (the bars show diffraction lines of anatase from ICDD card #00-021-1272 and brookite from ICDD card #00-029-1360).

**Figure 2:**
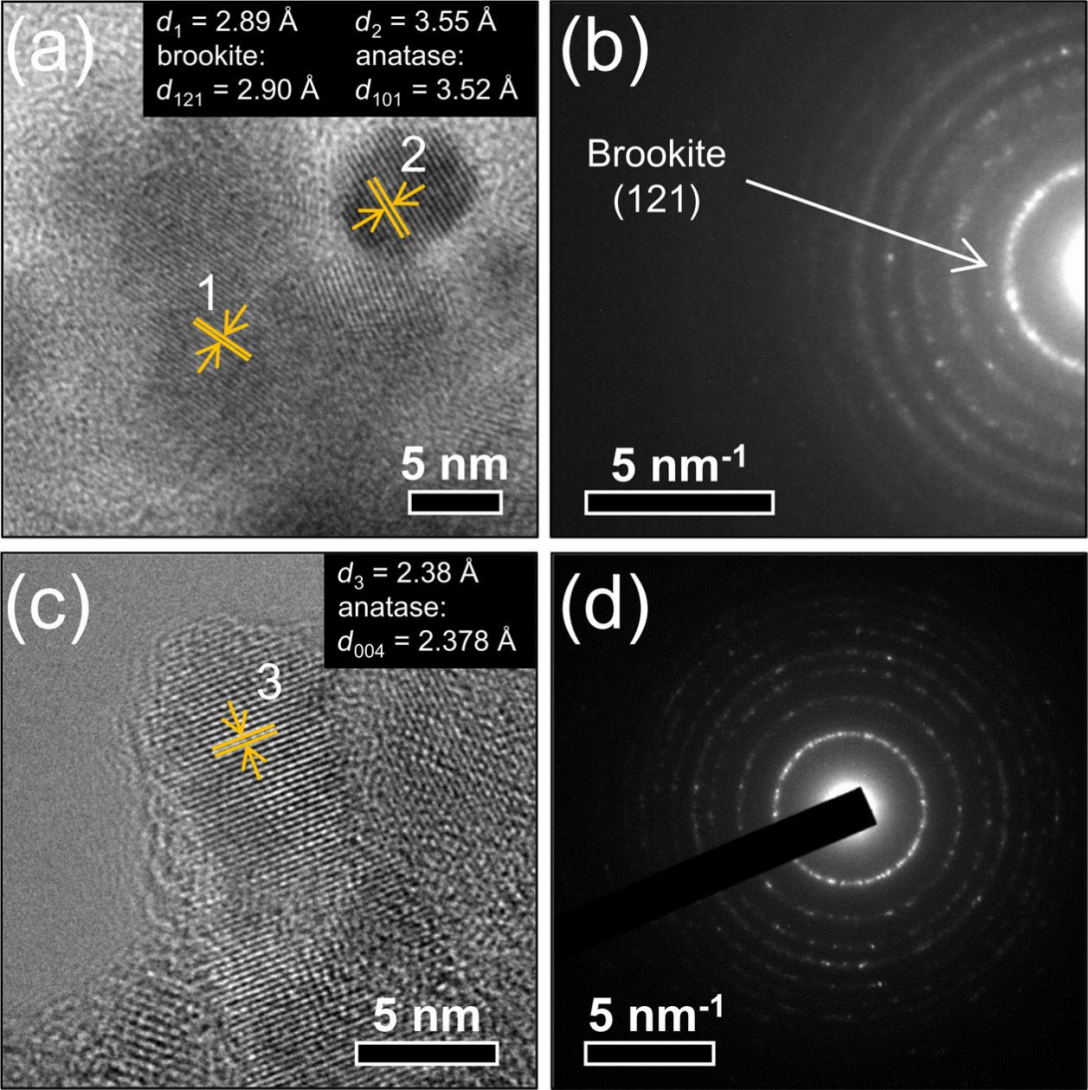
(a) HR-TEM image showing two individual nanoparticles of (1) brookite and (2) anatase and (b) electron diffraction pattern of pure TiO_2_ (top); (c) HR-TEM image of a rod-like nanoparticle and (d) electron diffraction pattern of Ti-APTES (bottom).

XRD patterns of TiO_2_ and in situ surface-functionalized TiO_2_ nanoparticles, presented in Figure 1, show anatase as main phase with around 25 wt % of brookite in the case of pure TiO_2_ and Ti-DTES samples (for Rietveld refinements see Figure S1 of Supporting Information File 1). The broad diffraction lines demonstrate small crystallite sizes, which were determined by refinement to be between 4.7 and 9.1 nm (Table 1). The HR-TEM image of the pure TiO_2_ sample (Figure 2a) demonstrates that anatase and brookite nucleate as individual monocrystalline nanoparticles. The electron diffraction patterns also show anatase and brookite in the case of TiO_2_ (Figure 2b) while Ti-APTES (Figure 2d) is purely anatase.

**Table 1:** Properties of TiO_2_ and in situ surface-functionalized TiO_2_ nanoparticles from nitrogen adsorption, XRD, SEM, and TGA analysis.

sample	*S*_BET_^a^ (m^2^·g^−1^)	*d*_BET_^b^ (nm)	*d*_BJH_^c^ (nm)	*d*_XRD_^d^ (nm)	*d*_SEM_^e^ (nm)	organic mass loss (%)	surface coverage (nm^−2^)

TiO_2_	195	7.9	7.9	5.7	9.0 ± 0.6	n/a	n/a
Ti-APTES	178	8.7	9.2	6.0	16.4 ± 1.4	5.8	3.4
Ti-AEAPS	149	10.3	11.8	9.1	20.9 ± 3.2	6.7	2.3
Ti-DTES	114	13.5	9.7	4.7	9.2 ± 0.9	10.8	4.0

^a^BET specific surface area from nitrogen adsorption measurements; ^b^particle size estimated from BET specific surface area; ^c^average pore diameter from BJH desorption calculations; ^d^crystallite size from Rietveld refinement of XRD measurements; ^e^particle size from SEM observations.

**Figure 3:**
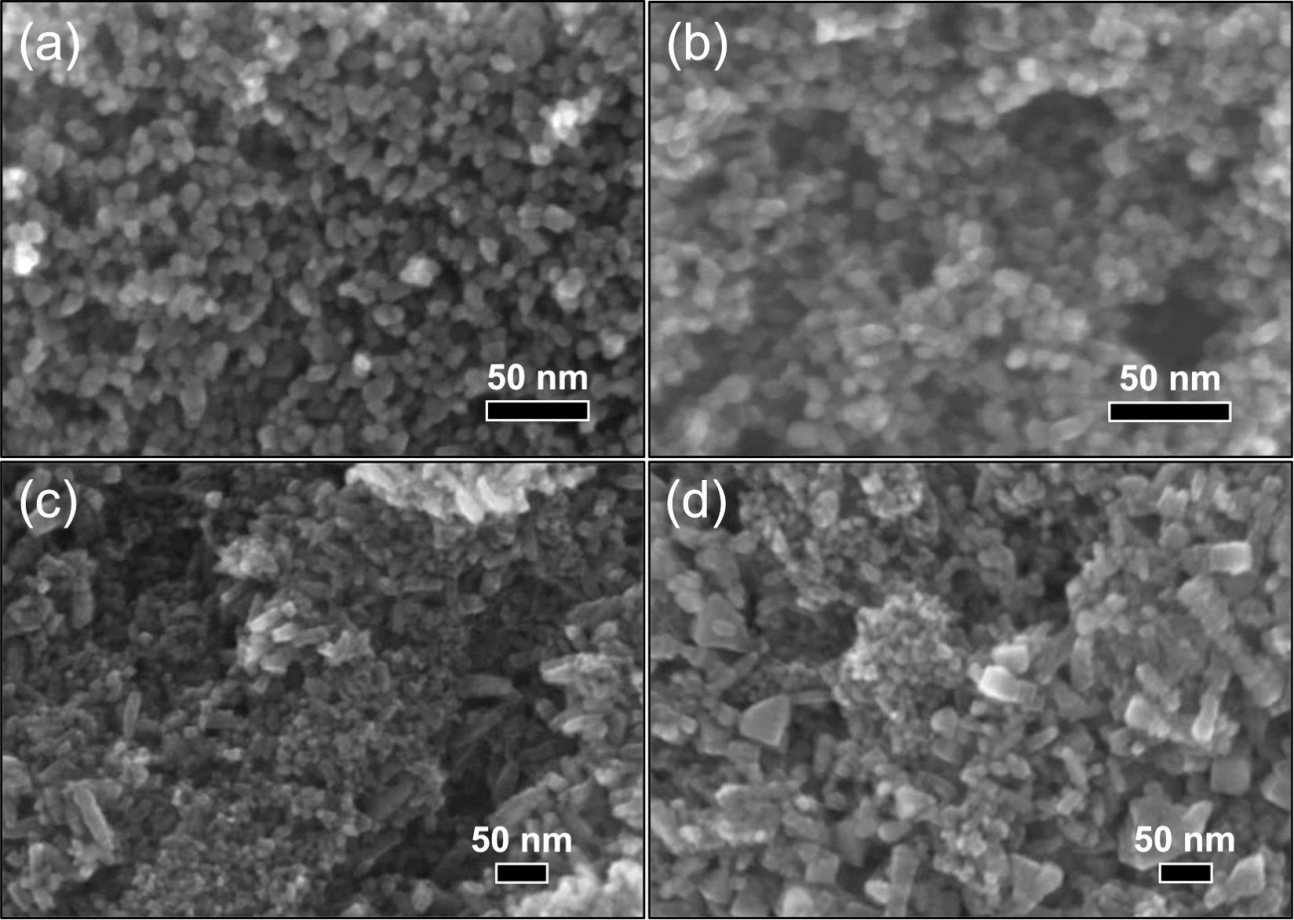
SEM images of (a) TiO_2_ and in situ surface-functionalized TiO_2_ nanoparticles, (b) Ti-DTES, (c) Ti-APTES, and (d) Ti-AEAPS.

SEM images of TiO_2_ and in situ surface-functionalized TiO_2_ nanoparticle agglomerates are displayed Figure 3. The measured particle sizes are included in Table 1. TiO_2_ (Figure 3a), and Ti-DTES (Figure 3b) samples consist of non-porous spherical nanoparticles with an average diameter of 9 nm and a narrow size distribution. Samples functionalized with aminosilane (Figure 3c,d) exhibit similar nanoparticles, but also larger rod-like nanoparticles. Similar crystallite size and roughness of the rod-like nanostructures suggest that they are formed by oriented attachment [31] of the nanoparticles. HR-TEM image of Ti-APTES (Figure 2c) shows {004} planes oriented perpendicularly to the elongation direction of a rod-like nanoparticle indicating growth along the [001] crystallographic direction, as previously reported for hydrothermally formed anatase [32]. This is also confirmed by the narrower FWHM of the (004) diffraction line at 37.80° (Figure 1 and Figure S1 of Supporting Information File 1) compared to other reflections. The rod-like nanoparticles are longer in the Ti-AEAPS sample (50–200 nm) compared to the Ti-APTES sample (50–100 nm) and they are not observed with the alkylsilane functionalization agent (DTES). Ahmad et al. [33] reported that different crystallographic faces of anatase exhibit different polarity and Kassir et al. [18] demonstrated that aminosilanes do not react homogeneously on the different faces of TiO_2_ nanoparticles. Thus, we propose that as the nanoparticles growth and functionalization occur simultaneously, rod-like nanoparticles originate from aminosilanes that guide the growth of the nanoparticles along the [001] crystallographic direction of anatase. The aminosilane-functionalized TiO_2_ nanoparticles are also the only ones that are purely anatase (Figure 1 and Figure S1 of Supporting Information File 1). Particle size and surface energy are some of the main factors for phase stability crossovers in nano-titania [5–7] and specific adsorption of aminosilanes could reduce the surface energy of the forming nanoparticles, promoting anatase nucleation during the synthesis, even if the crystallite and particle sizes are in this case larger than those measured for pure TiO_2_ (Table 1).

The measured BET specific surface area and the corresponding calculated size of the nanoparticles are included in Table 1. The particle sizes are consistent with the SEM and TEM observations and the crystallite sizes determined by XRD, which suggest only weakly agglomeration in the powders after drying.

**Figure 4:**
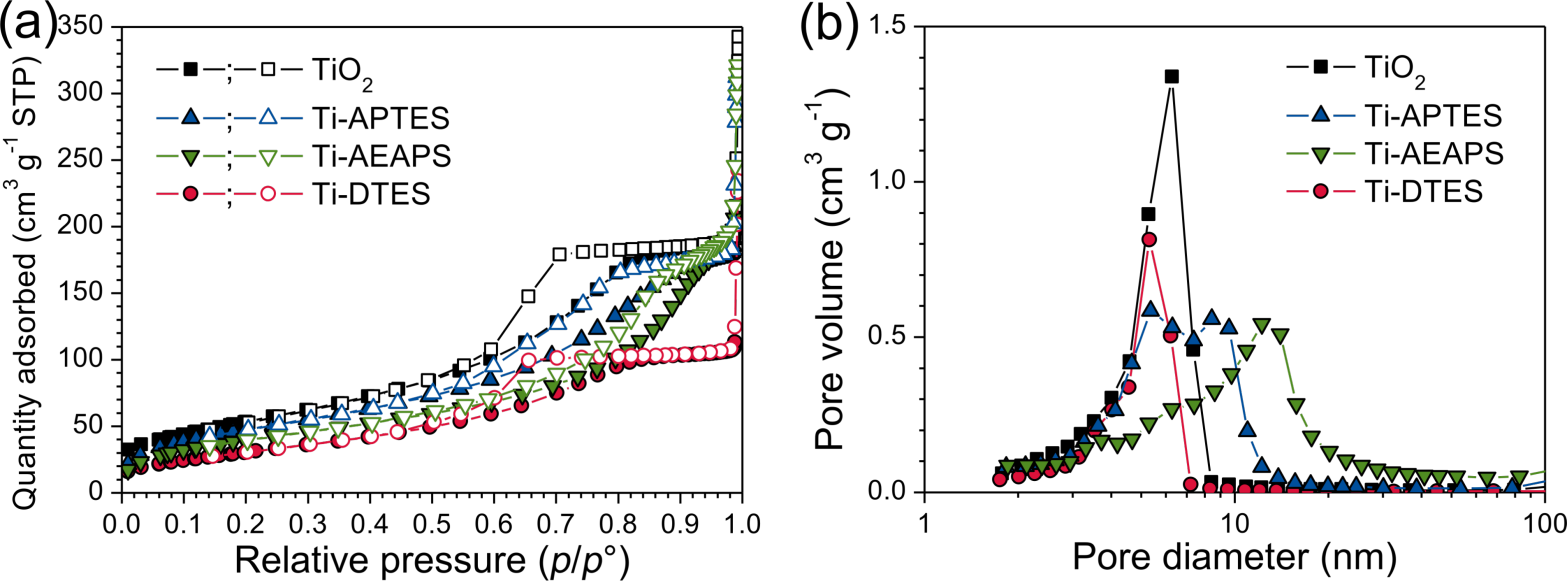
(a) Adsorption (solid symbols) and desorption (open symbols) isotherms and (b) BJH desorption d*V*/dlog(*D*) pore volume, from nitrogen adsorption measurements, of TiO_2_ and in situ surface-functionalized TiO_2_ nanoparticles.

The nitrogen adsorption and desorption isotherms of TiO_2_ and in situ surface-functionalized TiO_2_ nanoparticles demonstrate the hysteresis profile similar to mesoporous materials (Figure 4a). Since particle sizes from SEM and surface area are similar, porosity is associated with inter-particle volume of the agglomerates and can be directly correlated with the particle sizes [34]. Figure 4b displays the pore size distribution from desorption isotherms of TiO_2_ and in situ surface-functionalized TiO_2_ nanoparticles. The pore size distribution is centered between 4 and 7 nm for TiO_2_ and Ti-DTES, while for the aminosilane-functionalized samples, the size distribution is broader and shifted towards larger pores and two features are observed. The first feature centered between 4 and 7 nm is assigned to interstitial volume of the spherical nanoparticles and the second broader feature is assigned to interstitial volume of the rod-like particles. Coherently with SEM observations, as less spherical nanoparticles are observed in Ti-AEAPS, the volume of the feature between 4 and 7 nm is decreasing (relative comparison to Ti-APTES), and as rod-like particles are larger, the feature at 10 nm is shifted towards larger pores. The average pore diameters from BJH desorption curves are included in Table 1.

### Functionalization and hydrophobicity

**Figure 5:**
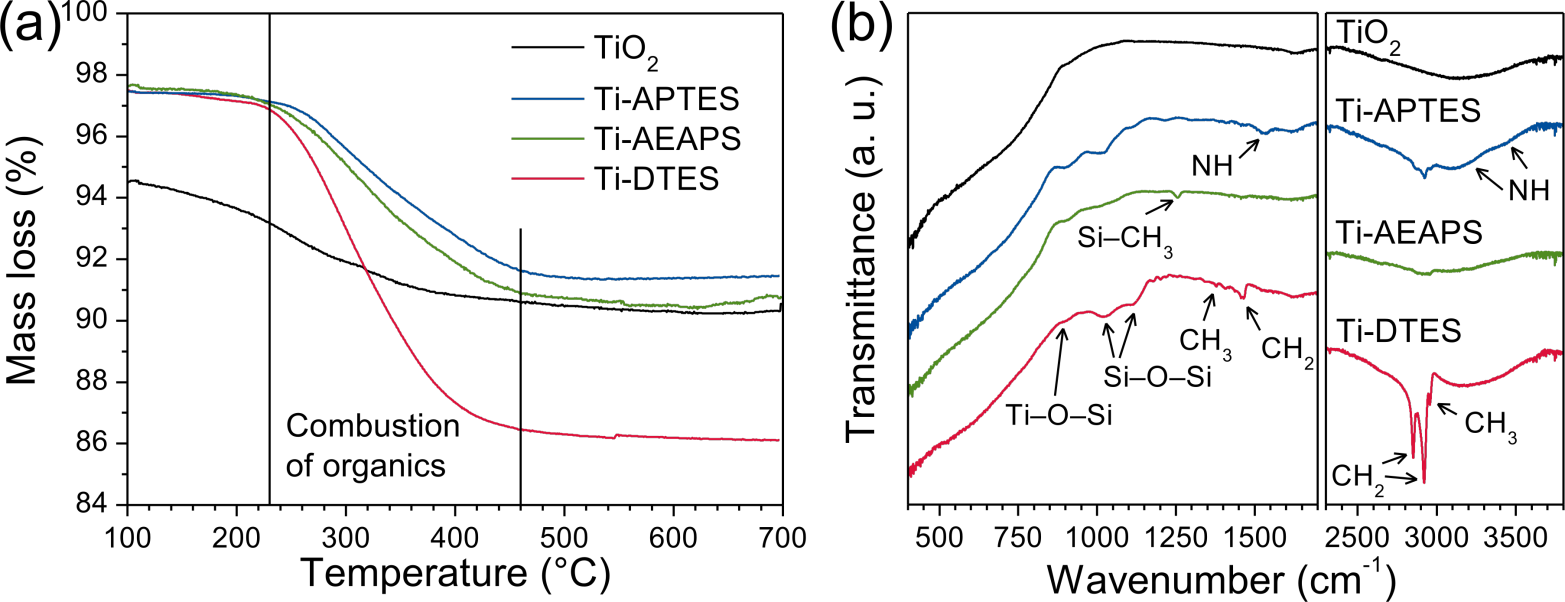
(a) TGA curves and (b) FTIR spectra of TiO_2_ and in situ surface-functionalized TiO_2_ nanoparticles.

Thermogravimetric analysis of TiO_2_ and the in situ surface-functionalized TiO_2_ nanoparticles are presented in Figure 5a. In case of pure TiO_2_, a significant mass loss assigned to hydroxy groups was observed until 400 °C. For the in situ surface-functionalized samples, the mass loss (at 230–460 °C) was assigned to the combustion of the organic part of the silane. The average surface coverages (molecules per square nanometer) of the nanoparticles were calculated based on the specific surface area and the mass loss due to combustion of the organic part, considering loss of C, H, and N of the silanes [35] (Table 1).

The FTIR spectra of TiO_2_ and in situ surface-functionalized TiO_2_ nanoparticles are shown in Figure 5b (see Table S1 of Supporting Information File 1 for the assignments of the absorption bands). No specific bands from isopropanol were observed indicating full reaction of the TIP precursor and high purity of the nanoparticles. In the case of in situ surface-functionalized samples, Si–O–Si bands at 1020 and 1120 cm^−1^ and the Ti–O–Si shoulder at 910 cm^−1^ confirmed that the silanes react via a condensation mechanism, cross-link, and covalently bond on the surface of the TiO_2_ nanoparticles [15–16,36]. The degree of order in the organic monolayer can be qualitatively estimated by comparing the CH_2_ stretching modes in crystalline (highly ordered) and liquid (highly disordered) states [36–37]. For Ti-DTES sample, the CH_2_ stretching modes at 2852 and 2921 cm^−1^ are close to those measured for crystalline polymethylene and for CH_3_(CH_2_)_9_SH adsorbed on gold [37] indicating well-ordered organic monolayers. In case of the aminosilane-functionalized samples, the low signal/noise ratios do not allow for an accurate measurement of the band positions. Additionally, because of possible surface contamination (from the carbon-coated grid) and/or optical aberration, the HR-TEM observation of Ti-APTES (Figure 2c) did not show clear evidence of the organic layer, confirming the nanometric nature of the organic coating.

**Figure 6:**
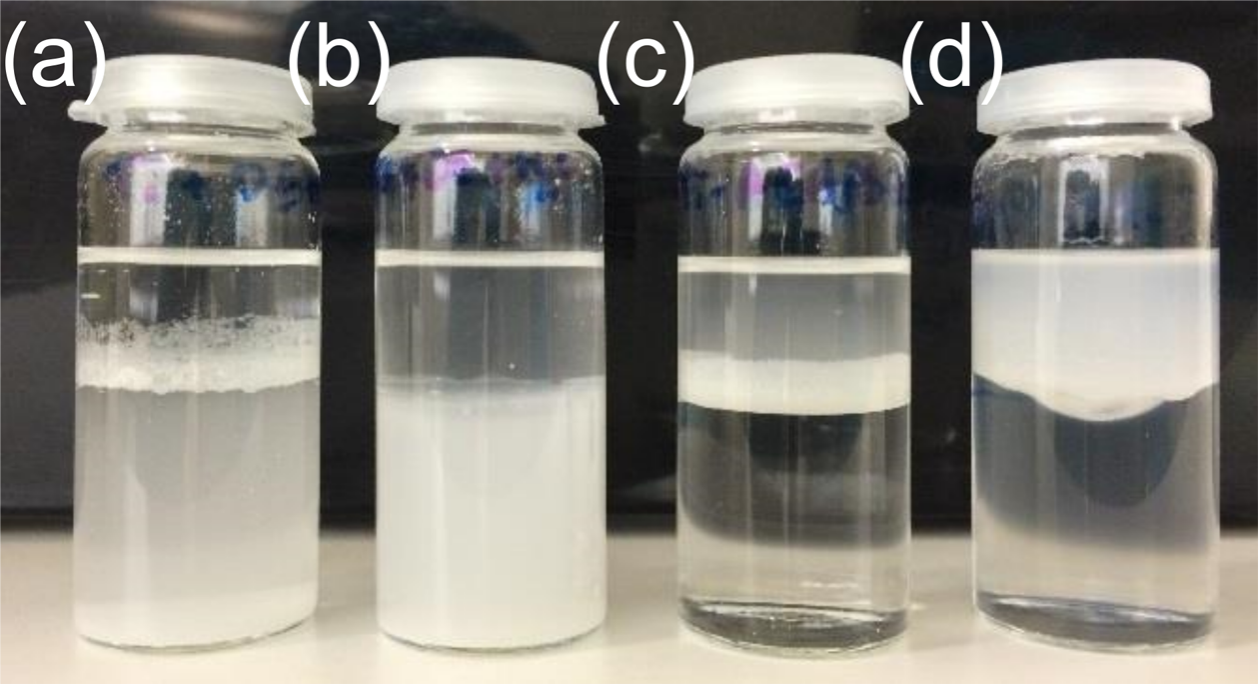
Photograph showing the behavior of (a) TiO_2_ and in situ surface-functionalized TiO_2_ nanoparticles, (b) Ti-APTES, (c) Ti-AEAPS, and (d) Ti-DTES in a mixed solutions of diethyl ether (top) and water (bottom).

A photo of TiO_2_ and in situ surface-functionalized TiO_2_ nanoparticles in a mixed solution of diethyl ether and water is displayed in Figure 6. Pure TiO_2_ entirely dispersed in the water phase and formed a blurred suspension, showing hydrophilic behavior. The Ti-APTES sample dispersed in both phases, indicating partial hydrophobic behavior. The Ti-AEAPS and Ti-DTES samples completely dispersed in the diethyl ether phase, demonstrating the hydrophobic behavior of these materials. The resulting hydrophobic properties of the nanoparticles depend on organic chain length and surface coverage [17], and are comparable to results reported by Iijima and co-workers on TiO_2_ nanoparticles post-modified with decyltrimethoxysilane and APTMS in mixed solutions of toluene and methanol [38].

### Heat treatment

**Figure 7:**
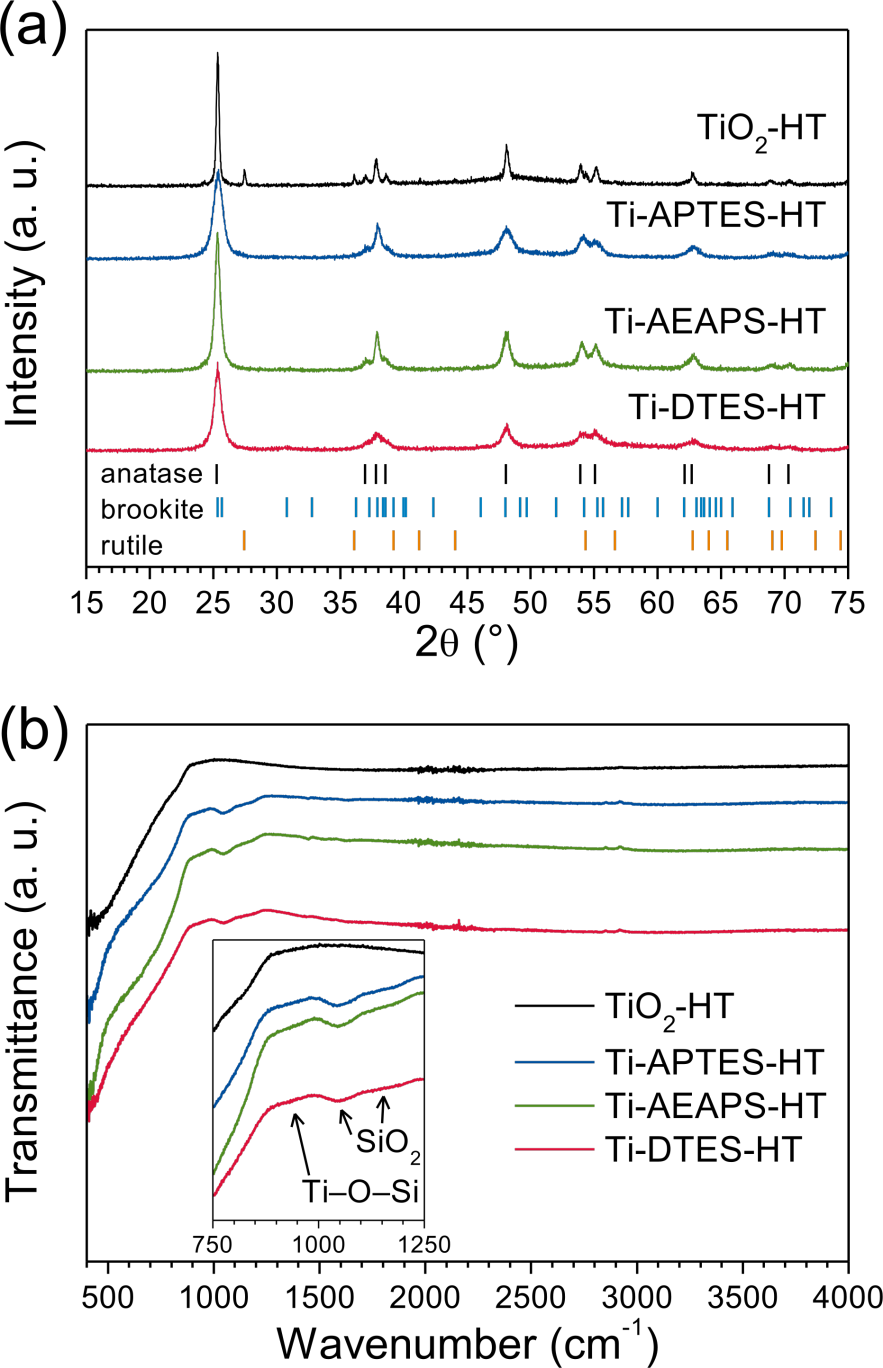
(a) XRD patterns (bars show diffraction lines of anatase from ICDD card #00-021-1272, brookite from ICDD card #00-029-1360, and rutile from ICDD card #00-021-1276) and (b) FTIR spectra of heat-treated TiO_2_ and in situ surface-functionalized TiO_2_ nanoparticles.

XRD patterns of heat-treated TiO_2_ and the in situ surface-functionalized TiO_2_ nanoparticles are shown Figure 7a, and these patterns were also refined (Figure S2 of Supporting Information File 1). While the pure TiO_2_ sample exhibits crystallites growth (from 5.7 to 28.8 nm), the functionalized nanoparticles with silane coupling agents showed only negligible growth of the crystallites after the heat treatment. Calculated crystallite sizes of the heat-treated samples are listed in Table 2. Additionally, in TiO_2_-HT, the heat treatment induced a partial phase transition from brookite and anatase to rutile, the thermodynamically stable polymorph of TiO_2_ [4]. In the case of in situ surface-functionalized TiO_2_ nanoparticles, the heat treatment has negligible effects on the crystallographic structure of the samples.

**Table 2:** Properties of heat-treated TiO_2_ and in situ surface-functionalized TiO_2_ nanoparticles from XRD and SEM analysis.

sample	*d*_XRD_^a^ (nm)	*d*_SEM_^b^ (nm)

TiO_2_-HT	28.8	30.0 ± 2.9
Ti-APTES-HT	7.8	15.5 ± 2.1
Ti-AEAPS-HT	10.4	23.7 ± 2.2
Ti-DTES-HT	8.9	12.0 ± 0.6

^a^crystallite size from Rietveld refinement of XRD measurements; ^b^particle size from SEM observations.

**Figure 8:**
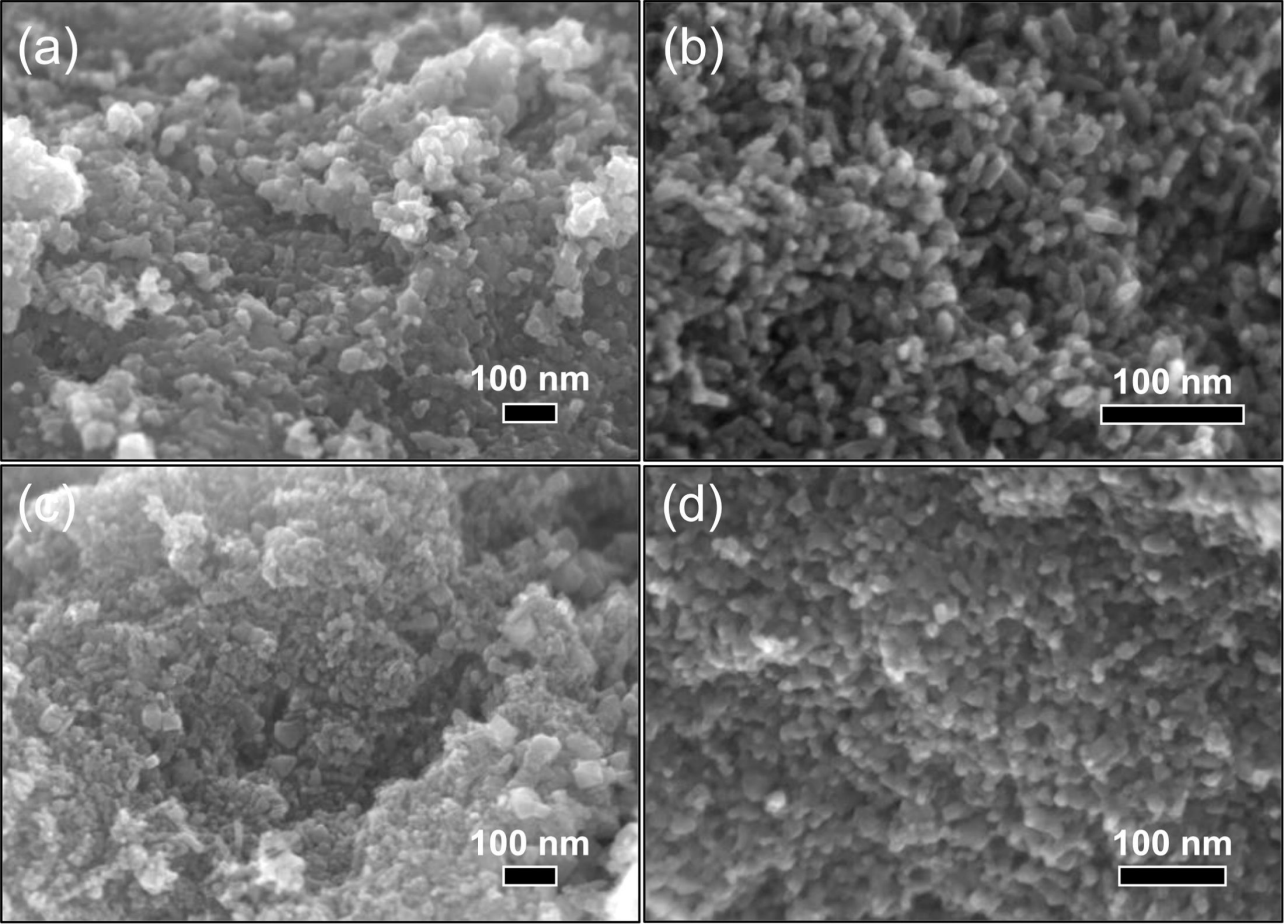
SEM images of (a) TiO_2_-HT and heat-treated in situ surface-functionalized TiO_2_ nanoparticles, (b) Ti-APTES-HT, (c) Ti-AEAPS-HT, and (d) Ti-DTES-HT.

The SEM images of heat-treated nanoparticles (Figure 8) confirmed the conclusions regarding both the morphology and the particles sizes (Table 2) showing the growth of the TiO_2_-HT nanoparticles.

The FTIR investigations of the heat-treated nanoparticles (Figure 7b) show absorption bands at 1050 and 1150 cm^−1^, which were assigned to Si–O–Si vibrations in silica [39] and a weak shoulder centered at 930 cm^−1^ was assigned to Ti–O–Si vibrations, in addition of the large absorption band below 900 cm^−1^ due to Ti–O–Ti vibrations.

**Figure 9:**
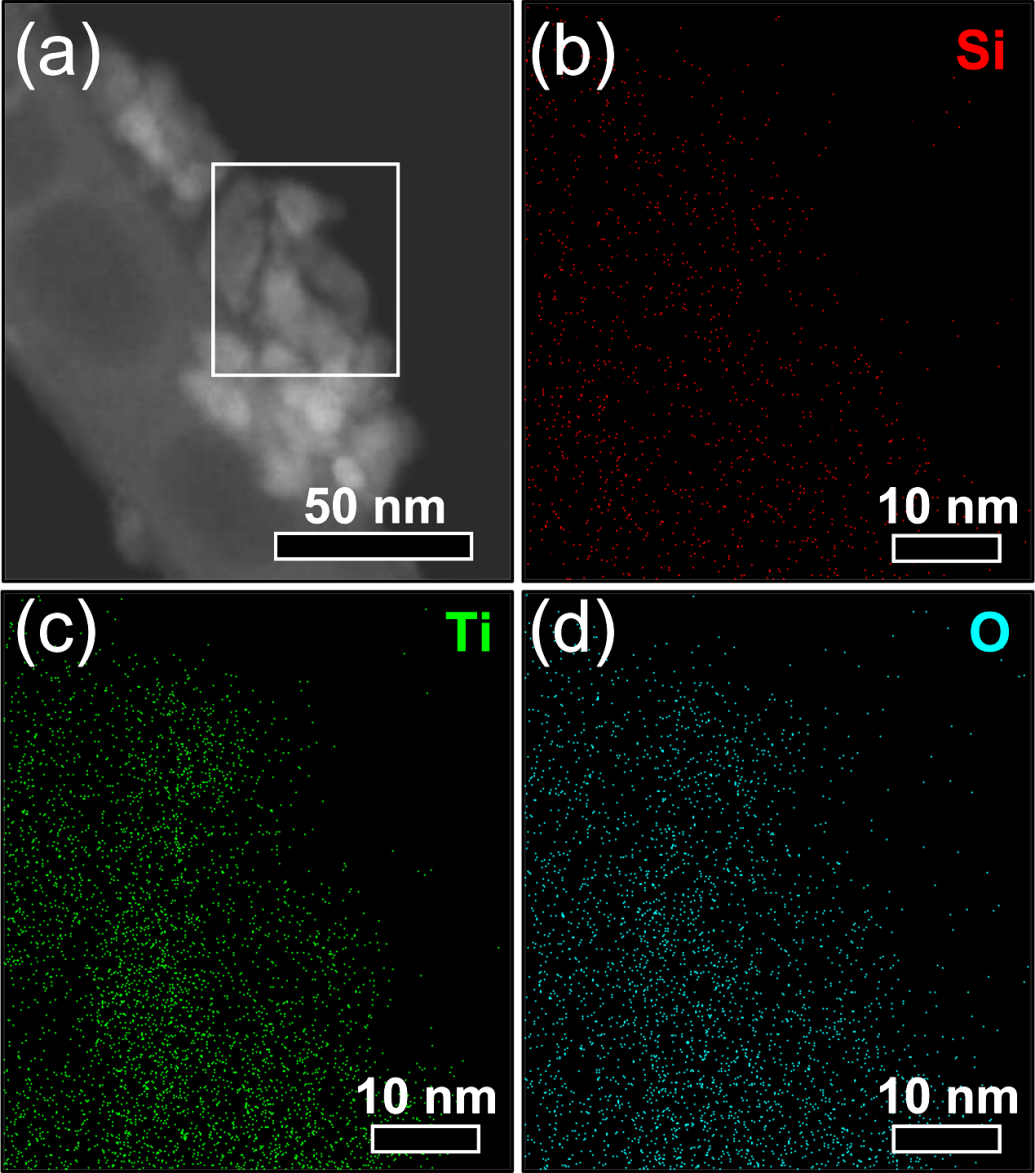
(a) Scanning electron image of Ti-APTES-HT with localization of the mapping (white rectangle) and EDS maps of (b) Si Kα_1_, (c) Ti Kα_1_, and (d) O Kα_1_ signals.

The EDS maps of the Ti-APTES-HT nanoparticles (Figure 9) show that silicon is homogeneously distributed over the particles. EDS spectra over relatively large areas of the heat-treated in situ surface-functionalized nanoparticles and the pure TiO_2_ samples (Figure S3 of Supporting Information File 1) confirm the presence and the absence of silicon, respectively.

Removal of the organic part of the silane coupling agent during the heat treatment induces the formation of a SiO_2_ layer on the surface of the TiO_2_ nanoparticles. The nature of the amorphous SiO_2_ layer could not be fully determined by TEM observation (Figure S4 of Supporting Information File 1), but it indicated nanometric thickness of the layer. The amorphous SiO_2_ layer inhibits surface diffusion of titanium, which prevents growth and phase transition of the nanoparticles, even at relatively high temperatures. Reduced crystallite growth and retarded phase transition have also been observed when firing mixtures of TiO_2_ and SiO_2_ powders [40].

## Conclusion

A new simple hydrothermal route to in situ surface-functionalized TiO_2_ nanoparticles has successfully been developed. Spherical hydrophobic TiO_2_ nanoparticles with a size of about 9 nm were prepared using silane coupling agents to functionalize the surface. Using aminosilane, the TiO_2_ nanoparticles showed oriented attachment along the [001] crystallographic direction of anatase to form rod-like nanostructures with a diameter close to the one of the spherical particles and a length in the range 50–200 nm dependent on the type of silane coupling agent. Surface coverage of the nanoparticles was measured to be between 2.3 and 4.0 molecules per square nanometer. The one-step aqueous synthesis reported here reduces time, the number of steps needed, and the complexity of production of surface-functionalized TiO_2_ nanoparticles. Despite the hydrothermal conditions, the synthesis is simple, robust, and reproducible. The numerous varieties of silane coupling agents offer versatility for tuning the surface properties of the TiO_2_ nanoparticles that are required for selected applications. Further modifications of the synthesis route are also possible for tuning the properties towards various types of applications. For example, applying the nanoparticles as filler in polymer nanocomposites, hydrophobicity is a parameter of utmost importance [10,27].

Heat treatment of the in situ surface-functionalized nanoparticles at 700 °C revealed neither crystallite growth nor phase transition of TiO_2_ because of the formation of an amorphous SiO_2_ layer, originating from the silane coupling agents, and leading to TiO_2_–SiO_2_ core–shell nanoparticles.

## Supporting Information

Supporting Information features Rietveld refinements of diffractograms of in situ functionalized and heat-treated nanoparticles, EDS spectra, additional TEM images, and assignments of the FTIR absorption bands.

File 1 Additional experimental data.
