# Supplementary material for: Functionalized TiO2 nanoparticles by single-step hydrothermal synthesis: the role of the silane coupling agents
Source: Beilstein J Nanotechnol. 2017 Jan 31;8:304–12. doi: 10.3762/bjnano.8.33 (PMC5301916; doi:10.3762/bjnano.8.33)
Supplement: File 1 — Additional experimental data. [file Beilstein_J_Nanotechnol-08-304-s001.pdf]

Supporting Information

for

Functionalized TiO_2 nanoparticles by single-step hydrothermal synthesis: the role of the silane coupling agents

Antoine R. M. Dalod[1], Lars Henriksen[2], Tor Grande[1] and Mari-Ann Einarsrud*,§1

Address: [1] Department of Materials Science and Engineering, NTNU, Norwegian University of Science and Technology, NO-7491 Trondheim, Norway and [2] poLight AS, Kongeveien 77, NO-3188 Horten, Norway

Email: Mari-Ann Einarsrud* - mari-ann.einarsrud@ntnu.no

*Corresponding author

§Telephone: +47 73 59 40 02

Additional experimental data

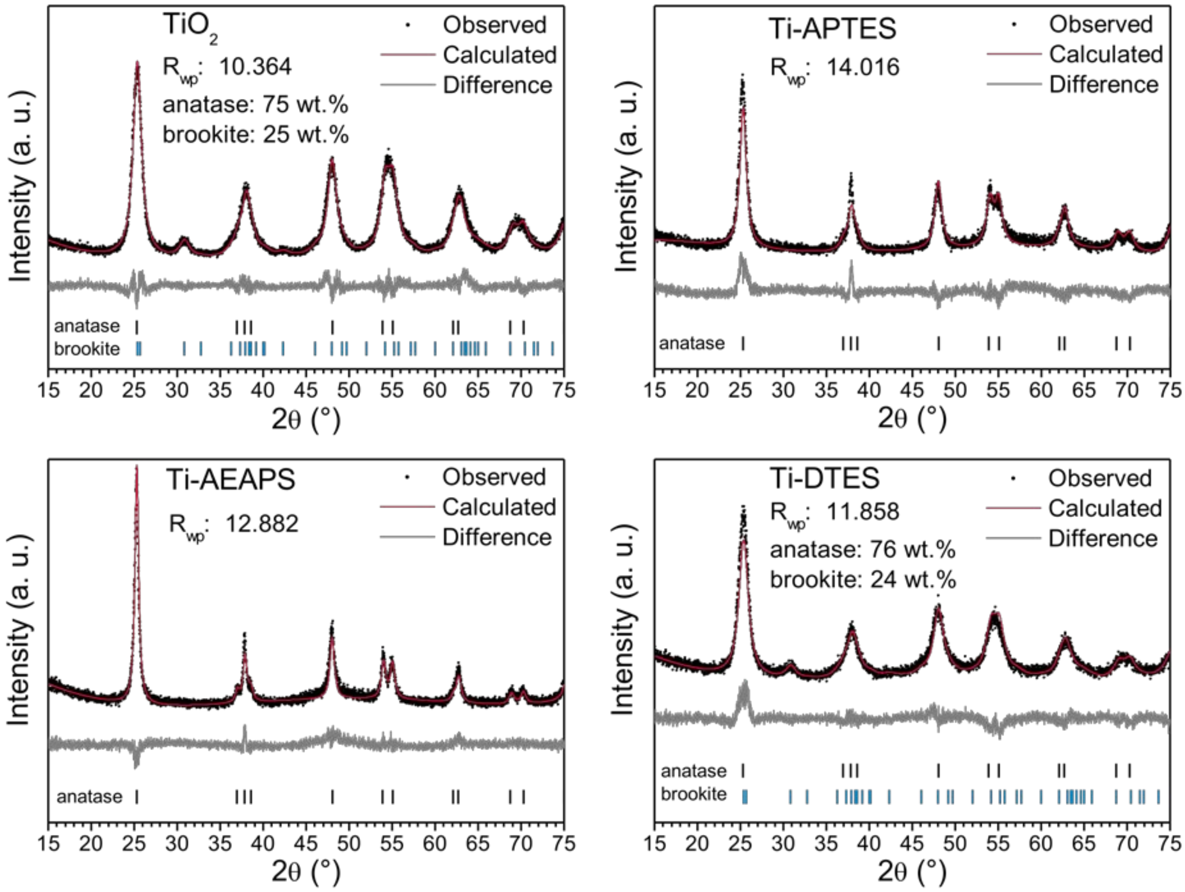

Figure S1: Rietveld refinements of TiO_2 and in situ surface-functionalized TiO_2 nanoparticles diffractograms (bars show diffraction lines of anatase from ICDD card #00-021-1272 and brookite from ICDD card #00-029-1360).

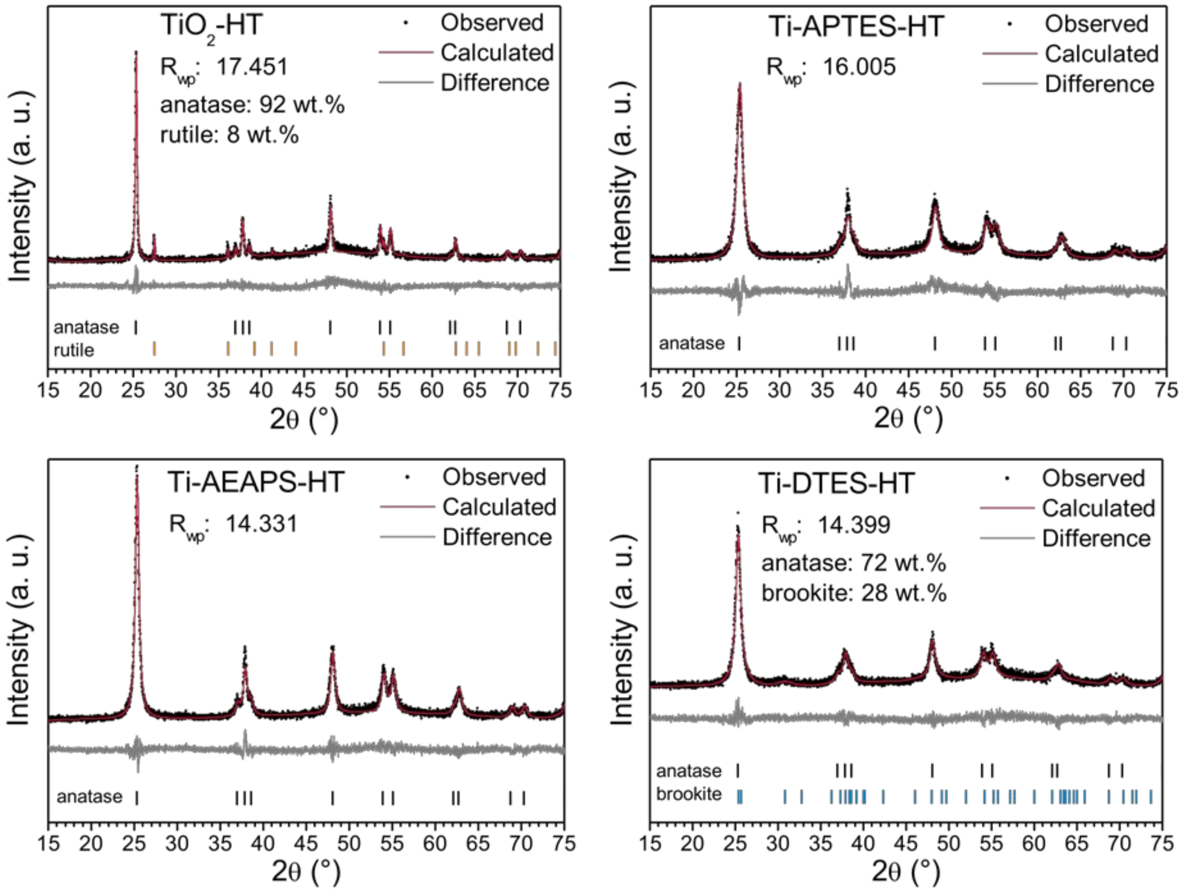

Figure S2: Rietveld refinements of heat treated TiO_2 and in situ surface-functionalized TiO_2 nanoparticles diffractograms (bars show diffraction lines of anatase from ICDD card #00-021-1272, brookite from ICDD card #00-029-1360, and rutile from ICDD card #00-021-1276).

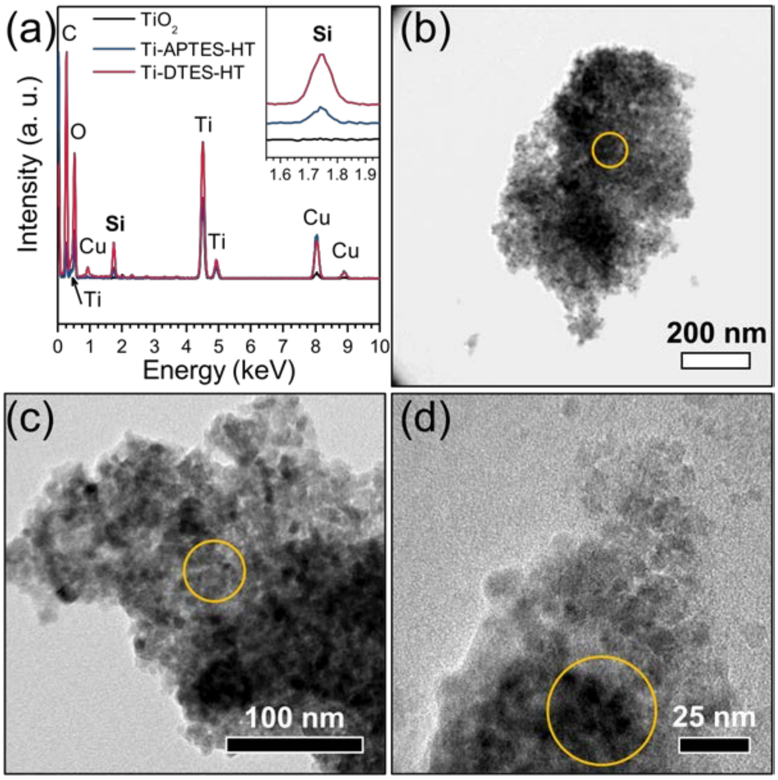

Figure S3: (a) EDS spectra from samples area (yellow circles) shown on TEM images of (b) TiO_2, (c) Ti-APTES-HT, and (d) Ti-DTES-HT, respectively.

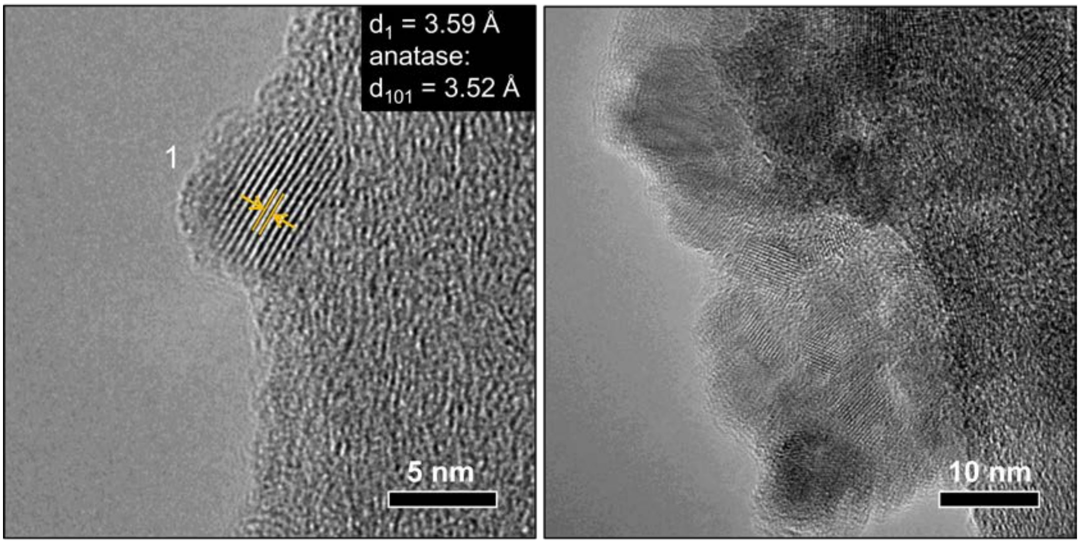

Figure S4: HR-TEM images of Ti-APTES-HT.

Table S1: Assignments of characteristic IR-bands of TiO_2 and *in situ* surface functionalized TiO_2 nanoparticles.

wave number (cm^{-1})	assignment	sample	references
<900	Ti–O–Ti	all	[1-4]
910	Ti–O–Si	all functionalized samples	[1-6]
1020 and 1120	Si–O–Si	all functionalized samples	[1-8]
1220	Si–CH_2R	all functionalized samples	[3,5,8]
1260	Si–CH_3	Ti-AEAPS	[3,5,9]
1380	CH_3 (deformation)	Ti-DTES	[3]
1460	CH_2 (deformation)	all functionalized samples	[3,8,10]
1530 and 3200–3500	N–H	Ti-APTES, Ti-AEAPS	[1,3,8,10]
1640 and 2500–3600	adsorbed H_2O and OH groups	all	[1,3,7,8]
2850 and 2920	CH_2 (stretching)	all functionalized samples	[1-3,8,11]
2870 and 2960	CH_3 (stretching)	Ti-DTES	[3,11]

References

[1] Zhao, J.; Milanova, M.; Warmoeskerken, M. M. C. G.; Dutschk, V. *Colloids Surf., A* **2012**, *413*, 273–279. doi:10.1016/j.colsurfa.2011.11.033

[2] Wang, C.; Mao, H.; Wang, C.; Fu, S. *Ind. Eng. Chem. Res.* **2011**, *50*, 11930–11934. doi:10.1021/ie200887x

[3] Socrates, G. *Infrared and Raman characteristic group frequencies: Tables and charts*; John Wiley & Sons: Chichester, UK, 2001.

[4] Koziej, D.; Fischer, F.; Kränzlin, N.; Caseri, W. R.; Niederberger, M. *ACS Appl. Mater. Interfaces* **2009**, *1*, 1097–1104. doi:10.1021/am9000584

[5] Matinlinna, J. P.; Laajalehto, K.; Lassila, L. V. J.; Yli-Urpo, A.; Vallittu, P. K. FT-IR and XPS surface characterization of allyltrimethoxysilane and 3-

methacryloxypropyltrimethoxysilane mixture adsorbed onto titanium substrate. In *Silanes and other coupling agents*; K. L. Mittal, Ed.; CRC Press: Boca Raton, FL, USA, 2004; Vol. 3, pp 21–37. doi:10.1201/b12184-5

[6] Milanesi, F.; Cappelletti, G.; Annunziata, R.; Bianchi, C. L.; Meroni, D.; Ardizzone, S. *J. Phys. Chem. C* **2010**, *114,* 8287–8293. doi:10.1021/jp1014669

[7] Chen, Q.; Yakovlev, N. L. *Appl. Surf. Sci.* **2010**, *257*, 1395–1400. doi:10.1016/j.apsusc.2010.08.036

[8] Plodinec, M.; Gajović, A.; Iveković, D.; Tomašić, N.; Zimmermann, B.; Macan, J.; Haramina, T.; Su, D. S.; Willinger, M. *Nanotechnology* **2014**, *25*, 435601. doi:10.1088/0957-4484/25/43/435601

[9] Czarnobaj, K. *Polym. Bull.* **2011**, *66*, 223–237. doi:10.1007/s00289-010-0332-8

[10] Iijima, M.; Kobayakawa, M.; Kamiya, H. *J. Colloid Interface Sci.* **2009**, *337*, 61–65. doi:10.1016/j.jcis.2009.05.007

[11] Porter, M. D.; Bright, T. B.; Allara, D. L.; Chidsey, C. E. D. *J. Am. Chem. Soc.* **1987**, *109*, 3559–3568. doi:10.1021/ja00246a011
